# Are Ki-67 and Procalcitonin Expression Levels Useful in Predicting the Biological Behavior of Hepatocellular Carcinoma After Liver Transplantation?

**DOI:** 10.3390/jcm14010144

**Published:** 2024-12-30

**Authors:** Ertugrul Karabulut, Sami Akbulut, Emine Turkmen Samdanci, Ayse Nur Akatli, Ahmed Elsarawy, Zeynep Kucukakcali, Zeki Ogut, Adem Tuncer, Volkan Ince, Sezai Yilmaz

**Affiliations:** 1 Department of Surgery and Liver Transplant Institute, Faculty of Medicine, Inonu University, 44280 Malatya, Turkey; 2 Department of Biostatistics and Medical Informatics, Faculty of Medicine, Inonu University, 44280 Malatya, Turkey; 3 Department of Pathology, Faculty of Medicine, Inonu University, 44280 Malatya, Turkey; 4 Department of Surgery, Gaziosmanpasa Hospital, 34245 Istanbul, Turkey

**Keywords:** hepatocellular carcinoma, procalcitonin, Ki-67 proliferation index, liver transplantation, recurrence, survival

## Abstract

**Background**: Examinations of procalcitonin (PCT) and Ki-67 expression levels in hepatocellular carcinoma (HCC) patients who have undergone liver transplantation (LT) through immunohistochemical analyses of tumor tissue may reveal the biological characteristics of the tumor, thus informing the selection of HCC patients for LT. **Methods**: Hepatectomy specimens from 86 HCC patients who underwent LT were obtained and analyzed immunohistochemically for the expression of PCT and Ki-67. The percentage and intensity of PCT staining, as well as the percentage of Ki-67 expression, were assessed for each patient. The impacts of PCT and Ki-67 expression on disease-free survival, overall survival, and the recurrence rate were studied, as well as their correlations with other clinicopathological features. **Results**: The recurrent HCC group showed a higher Ki-67 level (*p* < 0.001), larger maximum dominant tumor diameter (*p* < 0.001), and higher rate of vascular invasion (*p* = 0.001). The pre-transplant AFP (*p* = 0.001), maximum dominant tumor diameter (*p* < 0.001), number of tumor nodules (*p* < 0.001), rate of vascular invasion (*p* = 0.001), and Ki-67 level (*p* = 0.044) were higher in patients beyond the Milan criteria. Similarly, the pre-transplant AFP (*p* < 0.001); maximum dominant tumor diameter (*p* < 0.001); number of tumor nodules (*p* < 0.001); rates of portal vein tumor thrombus (*p* = 0.002), poor differentiation (*p* = 0.021), and vascular invasion (*p* < 0.001); and Ki-67 level (*p* = 0.010) were higher in patients beyond the expanded Malatya criteria. The maximum dominant tumor diameter (*p* = 0.006); Ki-67 level (*p* = 0.003); rates of vascular invasion (*p* < 0.001), cases beyond the Milan criteria (*p* = 0.042) and the expanded Malatya criteria (*p* = 0.027), and portal vein tumor thrombus (*p* = 0.020); and presence of recurrence (*p* < 0.001) were higher in HCC patients with mortality. The Kaplan–Meier estimates indicated that Ki-67 levels exceeding 5% significantly affected DFS and OS. Although the Kaplan–Meier estimates indicated that a PCT staining percentage of ≥25% did not have a statistically significant effect on DFS or OS, the outcomes may be considered clinically significant. **Conclusions**: This study demonstrated that the Ki-67 proliferation index can be used as a predictive biomarker of the biological behavior of HCC. Furthermore, we claim that PCT expression over a particular threshold might impact recurrence and survival, and we believe that further multicenter prospective studies focused on standardized PCT antibody staining are crucial in order to determine its potential as a biomarker for HCC.

## 1. Introduction

Primary liver cancer is a significant healthcare burden, being the sixth most common cancer worldwide and the third leading cause of all-cancer mortality [1,2,3], with hepatocellular carcinoma (HCC) representing 75–85% of primary liver cancers [4]. Liver transplantation (LT), within certain criteria, is the most common curative pathway of HCC treatment [1,3]. Since the adoption of the Milan criteria as a morphological surrogate of the clinicopathological behavior of HCC, many institutions have extended the allocation criteria or merged them with biological indicators, aiming at best-case selection [5]. Numerous LT criteria have been established to assess the tumor size, tumor number, standard histological characteristics, and tumor biology, either individually or together, with the goals of achieving an acceptable post-transplant recurrence rate and survival (both disease-free and overall) in patients with HCC. The most common LT criteria for HCC are the Paul-Brousse [6], Milan [5], UCSF [7], Up-to-seven [8], Asan [9], CUN (Navarra) [10], Valencia [11], Toso [12], Kyoto [13], Extended Toronto [14], Kyushu University [15], Onaca (ITR) [16], Samsung [17], Metroticket 2.0 model [18], alpha fetoprotein (AFP) model [19], Tokyo (5-5 rule) [20], BCLC [21], Shanghai [22], 5-5-500 rule [23], AFP-TTD [24], Malatya [25], and Expanded Malatya [26] criteria.

The recurrence of HCC following LT is a complex clinical situation, considering the immunosuppression state and the necessity of oncological intervention, as well as possible patient frailty or sustained long-term post-transplant complications. Therefore, identifying the cases that would benefit most from LT and concurrently carry the least risk of recurrence has been the focus of the LT scientific community over the last two decades [27]. Accordingly, immunohistochemical and biological predictors of HCC behavior have been heavily studied and introduced into the various selection criteria and prognostic nomograms. Chan et al. [28] indicated that numerous biomarkers have been defined for the diagnosis of HCC (e.g., tissue biopsy, liquid biopsy, imaging, ultrasound + AFP, serological biomarkers, circulating tumor cells, circulating tumor DNAs, extracellular vesicles, methylation, non-coding RNAs, viral load, omics technologies), determination of optimal therapeutic options, and evaluation of treatment response; particularly with regard to resection (recurrence, metastasis, AFP, AFP-L3, DCP, GP73, GPC3, CK19, PLT, miRNAs, lncRNAs, circRNAs, CTCs, EMT), liver transplantation (tumor size, tumor number, AFP, PET scan, viral load), TACE (drug resistance, survival, DNA-PKcs, VEGF, PKM2, cfDNAs, ctDNAs), targeted therapies (methylation, proteins, lncRNA, miRNA), and immunotherapies (drug resistance, survival, cytokines, immune cells, mutations, metabolomics, microbiome). A majority of the reported biomarkers are expensive and difficult to analyze routinely. Thus, it is essential to conduct patient-specific studies and concentrate on easily determined markers.

It has been suggested that the immunohistochemical biomarkers used in the context of HCC are linked to the biology and behavior of the tumor and, as a result, can be utilized as prognostic biomarkers. Nevertheless, it is widely recognized that their clinical application is restricted by the absence of a standardized immunohistochemical method. Based on this argument, Niu et al. [29] analyzed the data from some published studies and showed that over-expression of immunohistochemical markers such as Ki67, VEGF, MMP-2, MMP-9, CD44s, CD44v6, OPN, HMGB1, CD133, EpCAM, CK19, GPC3, CD105, and those associated with the mTOR pathway are independent prognostic factors for survival in patients with HCC. Wang et al. [30] have demonstrated that Ki67 and glypican are significant markers for predicting the biological behavior of HCC, and proposed that glypican may serve as a crucial target for immunotherapy in HCC. To the best of our knowledge, there is no research demonstrating the correlation between procalcitonin (PCT) and primary liver cancers, with the exception of one article [31] and several case reports [32,33,34,35,36,37,38]. Three published studies have demonstrated PCT expression in tumor tissue via immunohistochemical staining [32,36,38], whereas one study indicated elevated PCT gene expression via qRT-PCR technology [33]. The remaining four studies focused on the relationship between blood PCT levels and liver tumors.

PCT—a prohormone composed of 116 amino acids—is synthesized by parafollicular C cells of the thyroid gland and adipose tissue in healthy individuals, and it is subsequently delivered into the bloodstream for conversion into calcitonin to regulate serum calcium levels [39,40,41]. As it is synthesized in the parafollicular C cells of the thyroid gland, it is commonly used in the monitoring of patients with medullary thyroid carcinoma [41,42]. PCT is secreted by many parenchymal tissues in response to bacterial toxins, tumor necrosis factor alpha, and proinflammatory cytokines; hence, in clinical practice, PCT is recognized as a precise indicator of the severity of bacterial infection and the efficacy of antimicrobial treatment [39,40,41,43,44]. Some research findings have indicated that PCT serves as an effective biomarker for predicting post-hepatectomy liver failure [41,43]. PCT has been demonstrated to reach abnormal levels in cases of trauma, mechanical injury, burns, surgical procedures, and certain malignancies, including neuroendocrine tumors of the lung, gastrointestinal system, and pancreas [34]. Recently, elevated PCT levels have been observed in several cases of liver cancer, including HCC, cholangiocarcinoma, and hepatic neuroendocrine carcinoma [32,34,35,37]. Consequently, the immunohistochemical analysis of PCT as a molecular biological predictor of HCC behavior has raised attention regarding its utility as a potential biological marker; particularly, considering the persistent elevation of plasma PCT observed after preliminary treatment in some cases of HCC [32,36].

Ki-67—also known as MKI67 and MIB1—is one of the most widely studied indicators concerning the biological activity of tumors. Ki-67 is a nuclear protein that is directly related to the proliferation rate of cells. The fact that Ki-67 is highly expressed in the G1, S, G2, and mitosis phases of the cell cycle and absence of Ki-67 expression in the resting stage indicates that Ki-67 is a protein responsible for cell growth and proliferation [45,46]. The assessment of tumor proliferation activity with respect to Ki-67 levels has been investigated as an independent prognostic and predictive factor in several cancer studies, including HCC [29,47]. The primary objective of this study is to demonstrate the impacts of Ki-67 and PCT expression levels in cancer tissue on post-transplant recurrence and survival, utilizing clinical and oncological data, in order to determine the potential of Ki-67 and PCT as biomarkers.

## 2. Materials and Methods

### 2.1. Definition of the Study Population

Between April 2006 and October 2021, a total of 3041 LT procedures were performed at Inonu University Liver Transplant Institute, of which 396 involved HCC along with the underlying primary disease. Demographic, clinical, and follow-up data of 396 patients with HCC, referred to as the entire cohort, were prospectively recorded and retrospectively assessed to organize this study. First, the required number of HCC patients for the study was determined using the sample size calculation (G*Power 3.1.9.7), and it was concluded that the required number of patients should be 84 (two-tailed, effect size d = 0.4, alpha = 0.05, power = 95%, critical t = 1.99). Then, considering the unsuitable staining patterns on pathology slides and missing clinical data, together with the restricted budget allocated for the study, a sample of 100 HCC patients was chosen from the entire cohort. A random number generation method was used to objectively decide which HCC patients from the entire HCC cohort would be included in the study, and the R program was used for this purpose. Consequently, due to some staining technique-related problems, only 86 HCC patients could be successfully stained with both PCT and Ki-67 antibody, and these patients were included in the study. In order for the pathologists to make objective evaluations, the tumor characteristics, recurrence, and survival status were not shared with them. The following parameters regarding the HCC patients were analyzed: age (years), gender (male, female), BMI (kg/m^2^), Model For End Stage Liver Disease (MELD) score, Child score (A, B, C), underlying primary liver disease (HBV, HDV, HCV, cryptogenic, metabolic disease, ethanol, etc.), LT type (LDLT, DDLT), pre-transplant last AFP level, maximum dominant tumor diameter (MTD), number of tumor nodules, tumor differentiation (well, moderately, poorly), vascular invasion (microvascular, macrovascular), Milan criteria (within, beyond) [5], expanded Malatya criteria (within, beyond) [26,48], portal vein tumor thrombus (PVTT), overall survival (OS), disease-free survival (DFS), recurrence status, outcomes (alive, dead), Ki-67 proliferation index, percentage of tumoral area stained with PCT antibody, and intensity of PCT antibody staining.

### 2.2. Histopathological and Immunohistochemical Analysis

**Figure 1:**
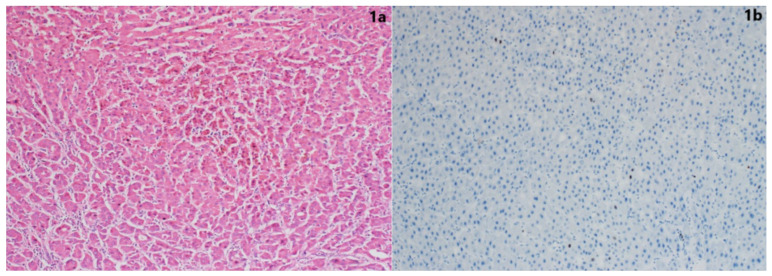

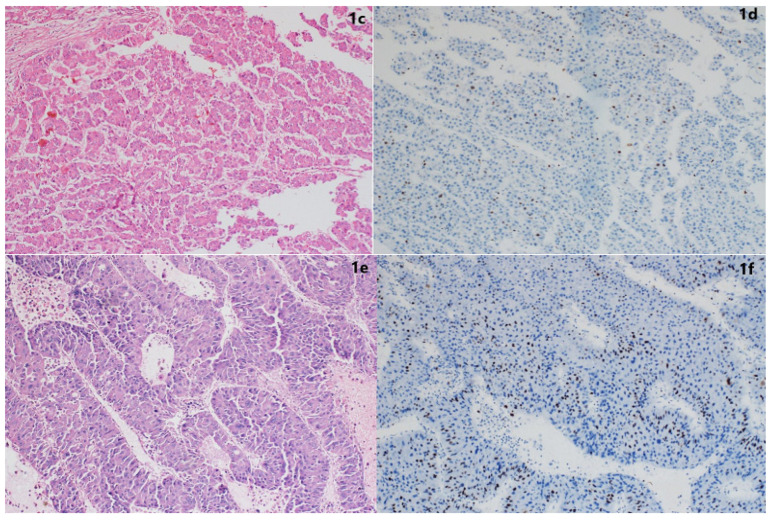
Staining patterns via histopathological differentiation: (**a**) Well-differentiated HCC (H&E ×10); (**b**) percentage of staining with Ki-67 antibody in well-differentiated HCC (positive staining in 5% of tumor cells) (Ki-67 antibody ×10); (**c**) moderately differentiated HCC (H&E ×10); (**d**) percentage of staining with Ki-67 antibody in moderately differentiated HCC (positive staining in 20% of tumor cells) (Ki-67 antibody ×10); (**e**) poorly differentiated HCC (H&E ×10); and (**f**) percentage of staining with Ki-67 antibody in poorly differentiated HCC (positive staining in 40% of tumor cells).

The hematoxylin and eosin sections of formalin-fixed and paraffin-embedded biopsies (hepatectomy explants) of the cases were retrospectively re-evaluated, and the tumor area was selected and included in the study. Necrotic and hemorrhagic areas within the tumoral area were excluded from the study. Three-micron-thick serial sections were cut and mounted on positively coated slides for immunohistochemical investigation. The immunohistochemical staining of the sections was performed using a fully automatic immunohistochemistry device (DAKO, Omnis). The primary antibodies used for the sections were Ki67 monoclonal antibody (Dako, ready-to-use) and PCT antibody (Abcam, Anti-PCT antibody [4A6], 1/150). PCT antibody staining was scored semi-quantitatively according to the percentage of the stained areas (no staining: 0, staining in 10% of the tumoral area: 1, staining in 25%: 2, staining in 25–50%: 3, staining in more than 50%: 4) and the intensity of staining (no staining: 0, weak staining: 1, moderate staining: 2, strong staining: 3) (Figure 1a–f).

**Figure 2:**
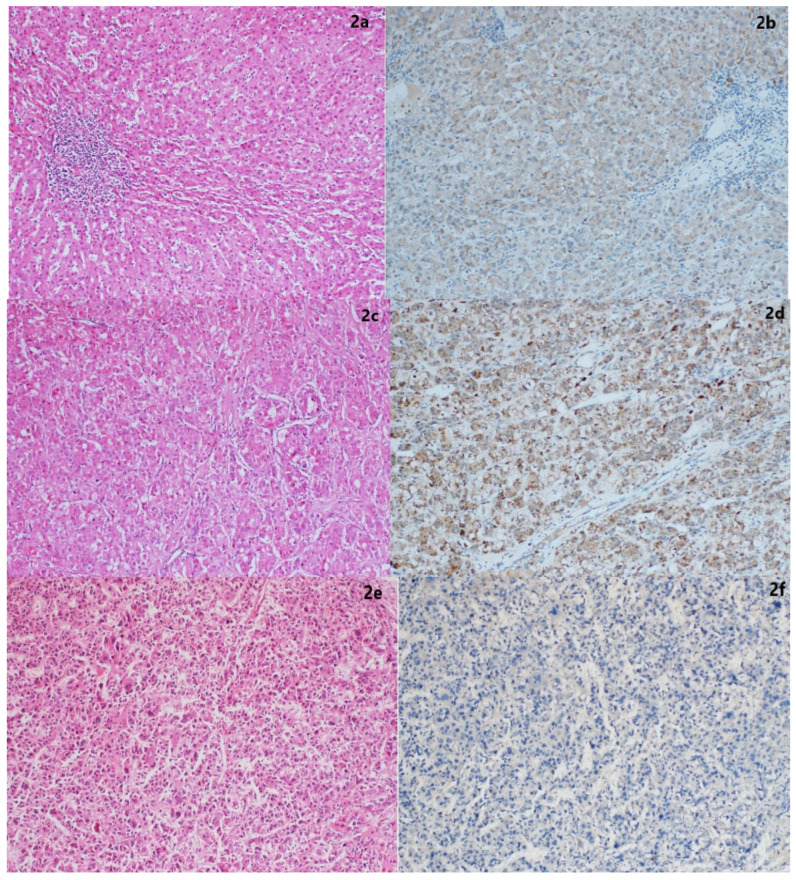
Staining patterns by histopathological differentiation: (**a**) Well-differentiated HCC (H&E ×10); (**b**) staining of tumor cells with PCT antibody (intensity of stained area, score: 2; severity of staining, score: 1) in well-differentiated HCC (PCT antibody ×10); (**c**) moderately differentiated HCC (H&E ×10); (**d**) staining of tumor cells with PCT antibody (intensity of stained area, score: 3; severity of staining, score: 3) in moderately differentiated HCC (PCT antibody ×10); (**e**) poorly differentiated HCC (H&E ×10); and (**f**) negative staining with PCT antibody in poorly differentiated HCC (PCT ×10).

Human tonsillar palatine tissue for Ki-67 antibody and thyroid medullary carcinoma for PCT antibody were used as positive external controls. Hematoxylin and eosin sections stained with Ki67 and PCT antibodies were evaluated under a light microscope. The hot spot method was used for Ki-67 scoring, reported as the percentage of positive cells. Hot spots were defined as areas in which Ki-67 staining was particularly high, relative to the adjacent areas (Figure 2a–f).

### 2.3. Study Protocol, Ethics Committee Approval, and Financial Support

This descriptive and analytic study involving human participants was conducted in accordance with the ethical standards of the institutional and national research committee and with the 1964 Declaration of Helsinki and its later amendments or comparable ethical standards. Ethical approval was obtained from the Inonu University Institutional Review Board (IRB) for non-interventional studies (Date: 15 June 2021, Number: 2202). Each participant gave verbal and written consent before the transplantation procedure. The STROBE (Strengthening the reporting of observational studies in epidemiology) guideline was utilized to assess the likelihood of bias and overall quality for this study [49]. The study was supported and funded by the Inonu University Scientific Research Projects Coordination Unit (Project No: TSA-2021-2667).

### 2.4. Statistical Analysis

The normality of the distribution of the data was checked using the Shapiro–Wilk test. Numerical data were tested using the Mann–Whitney test and are presented as the median [95% confidence interval (CI)]. Categorical data were tested using Fisher’s exact test or Pearson chi-square test, as appropriate, and are presented as the number (percentage). To identify the independent risk factors affecting recurrence and mortality, variables considered significant in univariate analyses (*p* < 0.05) were included into the logistic regression model. A *p*-value of <0.05 was considered statistically significant. The analyses were conducted using IBM SPSS 26.0 software.

## 3. Results

### 3.1. General Assessment

This study included 86 HCC patients who had undergone LT, comprising 74 (86.0%) males and 12 (14.0%) females, with a median age of 58 (95% CI = 55–58) years. The median (95% CI) values of the BMI, MELD score, and pre-transplant AFP level were 27 (25–28) kg/m^2^, 12 (11–14) points, and 15 (12–25) ng/mL, respectively. The most frequent underlying diseases were as follows: HBV (n = 52; 60.5%), HBV+HDV (n = 7; 8.1%), Cryptogenic (n = 11; 12.8%), HCV (n = 12; 13.9%), metabolic disease (n = 2; 2.3), and ethanol (n = 2; 2.3%). A total of 39 (45.3%) patients were in the Child A category, 24 (27.9%) were in the Child B category, and 23 (26.7%) were in the Child C category. LDLT was performed in 84 (97.7%) patients and DDLT in 2 (2.3%) patients. A total of 47 patients (54.7%) fulfilled the Milan criteria, and 61 patients (70.9%) met the expanded Malatya criteria. Post-transplant recurrence was observed in 12 (14.0%) patients during a median DFS of 1117 days (95% CI: 944–1600). Post-transplant mortality was reported in 14 patients (16.3%), with a median OS of 1230 days (95% CI: 1016–1642).

### 3.2. Results of Histopathological and Immunohistochemical Features

The median (95% CI) values of the MTD and number of tumor nodules were 30 (30–40) mm and 2 (2–3), respectively. A total of 17 (19.8%) patients had well-differentiated tumors, while 51 (59.3%) patients had moderately differentiated tumors, and the remaining 18 (20.9%) had poorly differentiated tumors. Microvascular invasion was detected in 40 (46.5%) patients, while 7 (8.1%) patients demonstrated microvascular invasion; no vascular invasion was observed in the remaining 39 (45.4%) patients. PVTT was determined in 16 (18.6%) HCC patients. Immunohistochemical examination of the obtained hepatectomy specimens indicated that the median Ki-67 proliferation index was 10 (95% CI = 10–20). In terms of PCT antibody staining percentages, 84 HCC tumor tissues exhibited positive staining (<10% = 9 patients, 10–25% = 8 patients, 25–50% = 13 patients, greater than 50% = 54 patients), while only 2 patients revealed negative staining. In terms of the intensity of PCT staining, 55 (64.0%) HCC tumor tissues exhibited strong staining, 19 (22.1%) displayed moderate staining, 9 (10.5%) were weak, and 3 (3.5%) showed no staining intensity.

### 3.3. Comparison of HCC Patients with and Without Recurrence

Table 1 and Table 2 provide data comparing the HCC patients with (n = 12) and without (n = 74) recurrence. HCC patients with recurrence showed a higher Ki-67 index (*p* < 0.001), higher MTD (*p* < 0.001), higher microvascular and macrovascular invasion (*p* = 0.001), and lower DFS (*p* < 0.001). Among the HCC patients with recurrence, 91.7% and 75% of patients were beyond the Milan criteria (*p* = 0.002) and expanded Malatya criteria (*p* = 0.001), respectively. Variables indicating a *p*-value < 0.05 in the univariate analysis were included in the multivariate analysis model. Logistic regression analysis indicated that Ki-67 index (*p* = 0.005; OR: 1.12; 95%CI: 1.03–1.22) and MTD (*p* = 0.024; OR: 1.02; 95%CI: 1.01–1.04) were independent risk factors for recurrence (Hosmer and Lemeshow test; Chi-square = 6.78; *p* = 0.560).

**Figure 3:**
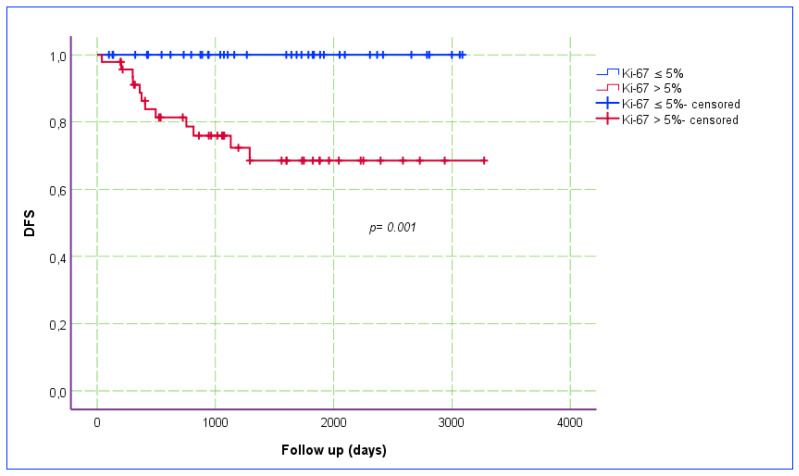
Kaplan–Meier estimate plot for the relationship between Ki-67 index and DFS.

**Figure 4:**
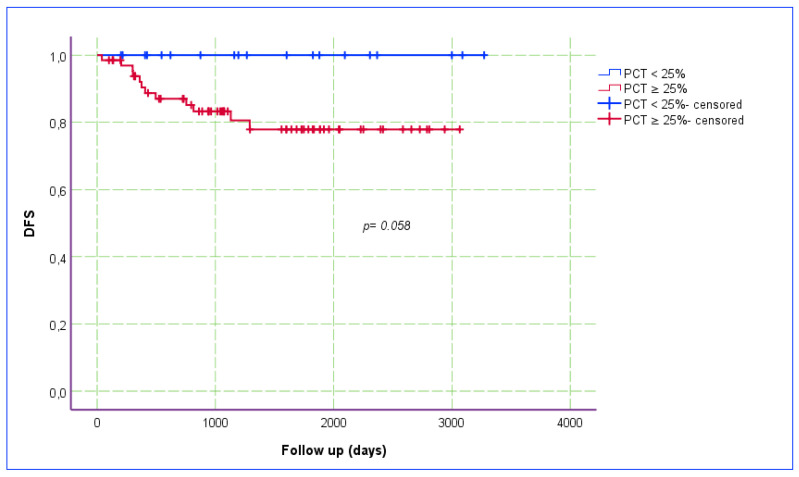
Kaplan–Meier estimate plot for the relationship between PCT staining and DFS.

The Ki-67 index was categorized into two groups, based on a cut-off point of 5% (i.e., ≤5% vs. >5%), and the relationship between the Ki-67 index and DFS was assessed using a Kaplan–Meier estimate plot (Figure 3). The recurrence rate was higher (OR: 14.4; *p* < 0.001) and DFS (*p* = 0.001) was poorer in patients with a Ki-67 index of >5%. The PCT staining pattern was categorized into two groups, based on the proportion of stained tumor area (<25% and ≥25%). The relationship between PCT staining and DFS was also assessed using a Kaplan–Meier estimation plot (Figure 4). Although the *p*-value was slightly above the threshold of statistical significance, DFS was notably superior in patients with a <25% PCT staining pattern.

### 3.4. Comparison of HCC Patients Within and Beyond the Milan Criteria

Table 3 and Table 4 provide data comparing the HCC patients within (n = 47) and beyond (n = 39) the Milan criteria. HCC patients who were beyond the Milan criteria showed a higher pre-transplant AFP level (*p* = 0.001), higher MTD (*p* < 0.001), higher number of tumor nodules (*p* < 0.001), lower DFS (*p* = 0.027), higher median Ki-67 index (*p* = 0.044), higher microvascular and macrovascular invasion (*p* = 0.001), higher mortality (*p* = 0.046), and higher recurrence (*p* = 0.002) than patients within the Milan criteria. In terms of the percentage and intensity of PCT staining, there were no statistically significant differences between the within and beyond Milan criteria groups in the current study.

### 3.5. Comparison of HCC Patients Within and Beyond the Expanded Malatya Criteria

Table 5 and Table 6 provide data comparing the HCC patients within (n = 61) and beyond (n = 25) the expanded Malatya criteria. Those beyond the expanded Malatya criteria showed higher pre-transplant AFP level (*p* < 0.001), higher MTD (*p* < 0.001), higher number of tumor nodules (*p* < 0.001), higher Ki-67 index (*p* = 0.010), lower OS (*p* = 0.006), lower DFS (*p* = 0.001), higher PVTT (*p* = 0.002), higher poor differentiation (*p* = 0.021), higher microvascular and macrovascular invasion (*p* < 0.001), higher mortality (*p* = 0.002), and higher recurrence (*p* = 0.001). There were no statistically significant differences between the within and beyond the expanded Malatya criteria groups in terms of the percentage and intensity of PCT antibody staining.

### 3.6. Comparison of HCC Patients with and Without Survival

Table 7 and Table 8 provide data comparing the HCC patients who were alive (n = 72) and dead (n = 14) at the end of the study. The dead group showed higher Ki-67 index values (*p* = 0.016), higher MTD (*p* = 0.011), lower DFS (*p* < 0.001), higher macrovascular invasion, more beyond the Milan criteria (*p* = 0.042), more beyond the expanded Malatya criteria (*p* = 0.027), and higher recurrence rate (*p* < 0.001). There were no statistically significant differences between the alive and dead groups in terms of the percentage (*p* = 0.214) or intensity (*p* = 0.700) of PCT antibody staining. Variables indicating a *p*-value below 0.05 in the univariate analysis were included in the multivariate analysis model. A logistic regression analysis indicated that recurrence (*p* = 0.021; OR: 7.35; 95%CI: 1.35–40.0) was an independent risk factor for mortality (Hosmer and Lemeshow test; Chi-square = 6.61; *p* = 0.579).

**Figure 5:**
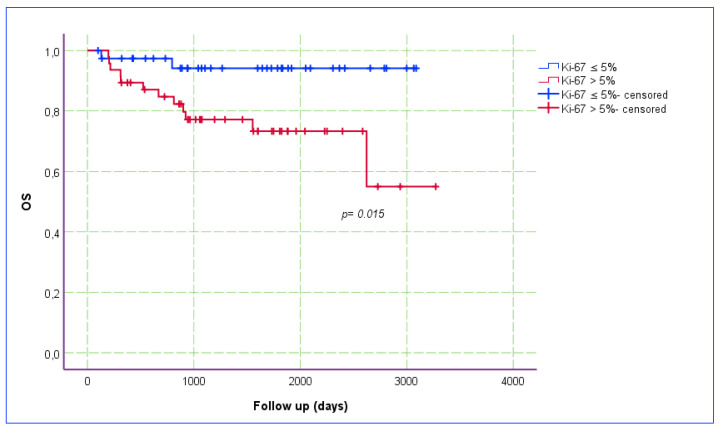
Kaplan–Meier estimate plot for the relationship between Ki-67 index and OS.

**Figure 6:**
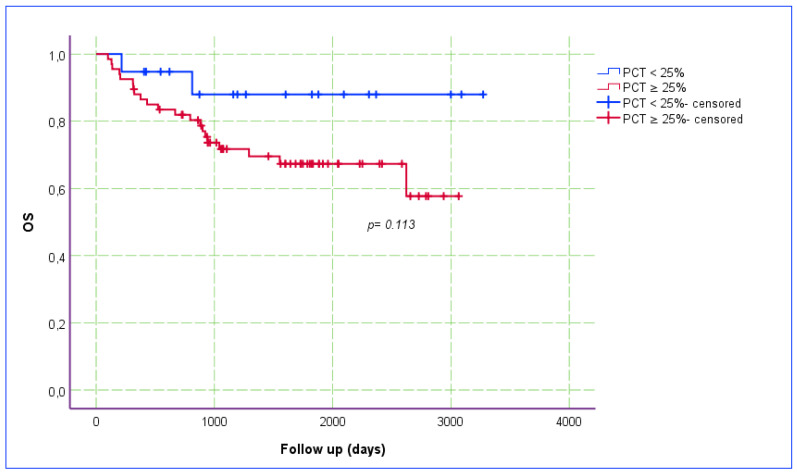
Kaplan–Meier estimate plot for the relationship between PCT staining and OS.

The Ki-67 index was categorized into two groups based on a cut-off point of 5% (i.e., ≤5% vs. >5%), and the relationship between the Ki-67 index and OS was assessed using a Kaplan–Meier estimate plot (Figure 5). The recurrence rate was higher (OR: 6.34; *p* = 0.017) and OS (*p* = 0.015) was poorer in patients with a Ki-67 index of >5%. The PCT staining pattern was categorized into two groups, based on the proportion of stained tumor area (<25% and ≥25%), and the relationship between PCT staining and OS was also assessed using a Kaplan–Meier estimation plot (Figure 6). Although the findings did not reach statistical significance (*p* = 0.113), OS outcomes appeared to be worse in patients with a ≥25% PCT staining pattern, which is important in terms of clinical significance.

## 4. Discussion

The implementation of LT for patients with HCC is the most definitive therapeutic option, as it allows for removal of the tumor combined with potentially cancerous liver tissue [50]. Despite the widespread usage of LT for HCC across both Eastern and Western medical centers, the recurrence rate remains relatively high, varying between 15 and 20% [51]. The recurrence of HCC after LT is a serious clinical consequence linked to reduced overall survival, with the median survival for patients who have HCC recurrence having been reported to be 10 to 13 months [52,53,54]. Since the initial implementation of the Milan criteria, which were designed to identify ideal candidates for LT, this field has experienced significant progress, focusing on optimizing the advantages of LT while minimizing the risk of recurrence [48]. Numerous morphological and biochemical characteristics of the tumor, which serve as indicators for post-transplant survival, have been utilized for patient selection to optimize survival. It is worth noting that, in the setting of LDLT, there is a continual necessity to enhance the selection criteria for HCC, as the brief waiting period may obscure aggressive tumor behaviors that would otherwise manifest over time. Most of the studies conducted to date have aimed to identify biomarkers that predict low tumor recurrence and long survival after LT, and to include these markers in LT selection criteria. This study attempted to predict the biological behavior of HCC through examining PCT and Ki-67 expression levels in liver tumor tissues.

The relationship between cancer and PCT—which is mainly secreted by the thyroid and adipose tissue in healthy individuals but is released by various parenchymal tissues under clinical conditions—remains scientifically controversial. It is known that, in patients with bacterial infection, PCT blood levels increase in response to toxins and some cytokines (TNF-α, IL-6, IL-1β), and its level decreases dramatically in parallel with the success of the treatment [55]. In cancer patients, an elevated level of PCT is associated with the tumor microenvironment, characterized by an inflammatory state where tumor cells and surrounding cells produce proinflammatory mediators such as TNF-α, IL-6, and IL-1 [55]. The significant decrease in PCT levels following treatment in cancer patients, along with the correlation between elevated PCT and poor prognosis, indicates that the fundamental underlying mechanism may be explained by the tumor microenvironment [31,32,38,56]. An analysis of the survival graphs in this study revealed that DFS and OS rates were superior in patients with a PCT expression level of <25%, when compared to those with higher PCT expression, indicating the clinical importance of this indicator.

Brunel et al. [33] reported a case of fibrolamellar HCC in a young adult presenting with acute abdomen alongside a ten-fold increase in PCT, which dropped significantly after a curative hepatectomy. Akbulut et al. [32] presented another report of a fibrolamellar HCC, which presented with high plasma PCT and underwent right hepatectomy with subsequent sharp decline in PCT levels. Though a significant difference in survival has been reported by Shen et al. when investigating 509 cases of irresectable HCC stratified according to the plasma PCT level [31], we did not obtain a similar level of significance in our study cohort when evaluating PCT at the cellular level. The explanation for this phenomenon is not yet clear but may be explained by the difference in clinical setting. The study of Shen et al. [31] likely involved a cohort with more aggressive tumor behavior than our patients, as evidenced by their higher median tumor diameter and higher median AFP levels. From an alternative viewpoint, the present study assessed the immunohistochemical expression of PCT in HCC liver tissue, whereas the research conducted by Shen et al. [31] evaluated PCT levels in blood. Consequently, a direct comparison between these two studies may be inappropriate. Moreover, standardization in blood studies is more readily achievable than when utilizing tissue staining techniques. The present study is, to our knowledge, the first and largest scientific study in the medical literature assessing PCT expression in liver tissue affected by HCC. Consequently, we claim that this study requires confirmation through prospective and multicentric studies conducted using comparable methods.

On the other hand, the present study revealed the significant association between a higher Ki-67 index in the HCC patients who were beyond multiple LT allocation criteria, being consequently associated with a higher incidence of post-transplant HCC recurrence and worse survival outcomes. This finding is consistent with the results of a meta-analysis (54 studies, 4996 patients) performed by Luo et al. [57], who revealed that HCC cases with high Ki-67 index were associated with higher grades, larger tumors, higher incidence of venous invasion, and distant metastases. The main difference between this meta-analysis and our study is that, in their pooled analysis, Luo et al. did not find a significant correlation between high Ki-67 index and poor survival outcomes in the subgroup of patients who underwent LT. In our study, statistically significant differences in terms of recurrence-free survival and overall survival were obtained in our cohort, which was an LT-only cohort. Therefore, it can be concluded that Ki-67 is a useful prognostic indicator in the LT setting.

Our results also aligned with a previous report by El-Gendy et al. [58], who correlated the percentage of Ki-67 immunohistochemical expression in a cohort of 114 cases of HCC who underwent partial hepatectomy or liver transplantation. The percentage of Ki-67 expression was positively correlated with three adverse tumor characteristics: larger MTD, more advanced stage, and older age.

Interestingly, our study demonstrated that Ki-67 staining was more significant in recurrent cases than AFP, which is routinely tested in the immediate LT interval. In view of this finding, Ki-67 staining in the pre-LT selection process interval—extracted from either a previous hepatectomy or pre-LT biopsy—may be a fruitful biological marker that provides insight into post-LT survival outcomes and, hence, could help in the decision-making process. At present, it is well acknowledged that sole dependence on AFP is not always precise for all clinical scenarios, as 30–40% of cases are almost AFP negative [59].

In order to avoid the heterogeneity—and, hence, inaccuracy—of immunohistochemical analysis, Ramos-Santillan et al. [60] studied the expression of the gene responsible for Ki-67 (MKi-67 gene) among 473 HCC samples and observed that higher gene expression correlated well with survival parameters, disease stage and progression, adverse histological criteria, and tumor microenvironment proliferative characteristics. Moreover, the authors observed no association between intra-tumoral immune response among HCC cases with higher gene expression [60]. In this study, there was no mention whether the HCC was managed by partial hepatectomy or LT; thus, it could inspire the institutions practicing LT to conduct similar explorations in a homogenous LT cohort.

This study has some limitations. First, the study was retrospective, and the sample size was limited. The high cost of Ki-67 and PCT antibody staining meant that the study sample had to be kept small. In addition, the fact that these analyses are not used in routine pathological examinations—especially the use of PCT staining—may lead to technical problems and failure to achieve standardization. In order to overcome these problems, prospective and multicentric studies that allow tissues to be analyzed at an early stage should be organized, and standardized procedures for staining should be developed. Second, the study presented here did not include the blood PCT levels of the patients. In order to overcome this problem, prospective studies should be organized and both pre-operative blood PCT levels and tissue staining patterns should be examined in patients, thus allowing for analyses to determine whether there exists a correlation between blood and tissue PCT levels.

## 5. Conclusions

This study demonstrated that Ki-67 expression is an independent prognostic factor for recurrence and survival, achieving both clinical and statistical significance. Therefore, we declare that Ki-67 can be used as a prognostic biomarker indicating the biological behavior of HCC. Despite the lack of statistical significance in the present study, the DFS and OS survival graphs indicated that PCT expression was also clinically significant concerning recurrence and survival. Finally, we believe that further multicentric prospective studies focused on standardized PCT antibody staining are crucial in order to determine its potential as a biomarker for HCC.

## Figures and Tables

**Table 1:** Analyses of quantitative variables in terms of recurrence variable categories.

Variables [Median (95%CI)]	HCC Recurrence		*p*-Value *
	No	Yes	
Age	58 (58–62)	52 (48–60)	0.079
BMI	27 (26–28)	25.7 (22–28)	0.295
MELD Score	12 (11–15)	10.55 (9–14)	0.146
Pretransplant last AFP level	14 (11–25)	26.5 (9–1001)	0.075
MTD (mm)	30 (30–40)	65 (40–150)	<0.001
Number of tumor nodules	2 (2–3)	3.5 (1–11)	0.117
OS	1426 (1055–1748)	911 (376–1552)	0.092
DFS	1426 (1055–1748)	391 (300–815)	<0.001
Ki67 Staining (%)	5 (5–10)	20 (20–30)	<0.001

* Mann–Whitney U-test; CI: Confidence Interval; BMI: Body mass index; MELD: Model For End-Stage Liver Disease; AFP: alpha fetoprotein; MTD: maximum dominant tumor diameter; OS: Overall survival; DFS: Disease-free survival.

**Table 2:** Analyses of qualitative variables in terms of recurrence variable categories.

Variables [n (%)]	Categories	HCC Recurrence		*p*-Value
		No	Yes	
Gender	Male	64 (86.5)	10 (83.3)	0.672 *
	Female	10 (13.5)	2 (16.7)	
Child Score	A	32 (43.2)	7 (58.3)	0.580 **
	B	21 (28.4)	3 (25.0)	
	C	21 (28.4)	2 (16.7)	
Etiology	HBV	45 (60.8)	7 (58.3)	0.624 **
	HBV + HDV	7 (9.5)	0 (0.0)	
	Cryptogenic	8 (10.8)	3 (25.0)	
	HCV	10 (13.5)	2 (16.7)	
	Metabolic Disease	2 (2.7)	0 (0.0)	
	Ethanol	2 (2.7)	0 (0.0)	
LT Type	LDLT	73 (98.7)	11 (91.7)	0.261 *
	DDLT	1 (1.3)	1 (8.3)	
Differentiation	Well	16 (21.6)	1 (8.3)	0.136 **
	Moderately	45 (60.8)	6 (50.0)	
	Poorly	13 (17.6)	5 (41.7)	
Vascular invasion	No	39 (52.7)	0 (0.0)	0.001 **
	Microvascular	31 (41.9)	9 (75.0)	
	Macrovascular	4 (5.4)	3 (25.0)	
Milan Criteria	Within	46 (62.2)	1 (8.3)	0.002 ***
	Beyond	28 (37.8)	11 (91.7)	
Expanded Malatya Criteria	Within	58 (78.38)	3 (25.00)	0.001 *
	Beyond	16 (21.62)	9 (75.00)	
PVTT	No	62 (83.8)	8 (66.7)	0.224 *
	Yes	12 (16.2)	4 (33.3)	
Outcomes	Alive	67 (90.5)	5 (41.7)	<0.001 *
	Exitus	7 (9.5)	7 (58.3)	
Ki-67	≤5%	39 (52.7)	0 (0)	<0.001 *
	>5%	35 (47.3)	12 (100)	
Percent of PCT stained tumoral area	No-staining	2 (2.7)	0 (0.0)	0.186 **
	<10%	9 (12.2)	0 (0.0)	
	10–25%	8 (10.8)	0 (0.0)	
	25–50%	9 (12.2)	4 (33.3)	
	>50%	46 (62.2)	8 (66.7)	
Intensity of PCTstaining	No	3 (4.0)	0 (0.0)	0.430 **
	Weak	9 (12.2)	0 (0.0)	
	Moderate	15 (20.3)	4 (33.3)	
	Strong	47 (63.5)	8 (66.7)	

*: Fisher’s exact chi-square test; **: Pearson chi-square test; ***: Chi-square test with Yates correction; HBV: Hepatitis B virus; HDV: Hepatitis D virus; HCV: Hepatitis C virus; LT: Liver transplantation; LDLT: Living donor liver transplantation; DDLT: Deceased donor liver transplantation; PVTT: Portal vein tumor thrombus.

**Table 3:** Analyses of quantitative variables in terms of Milan criteria.

Variables [Median (95%CI)]	Milan Criteria		*p*-Value *
	Within	Beyond	
Age	58 (57–62)	56 (53–59)	0.658
BMI	26 (25–28)	27 (25–28)	0.876
MELD Score	12 (11–15)	11 (10–14)	0.969
Pretransplant last AFP level	11 (5–14)	25 (17–75)	0.001
MTD	20 (20–30)	50 (40–70)	<0.001
Number of tumor nodules	1 (0–0)	4 (3–8)	<0.001
OS	1559 (1055–1879)	1104 (900–1602)	0.166
DFS	1559 (1055–1879)	938 (537–1292)	0.027
Ki67 Staining (%)	5 (5–10)	10 (10–20)	0.044

* Mann–Whitney U-test; CI: Confidence Interval; BMI: Body mass index; MELD: Model For End-Stage Liver Disease; AFP: alpha fetoprotein; MTD: maximum dominant tumor diameter; OS: Overall survival; DFS: Disease free survival.

**Table 4:** Analyses of qualitative variables in terms of Milan criteria.

Variables [n (%)]	Categories	Milan Criteria		*p*-Value
		Within	Beyond	
Gender	Male	39 (83.0)	35 (89.7)	0.556 *
	Female	8 (17.0)	4 (10.3)	
Child Score	A	25 (53.2)	14 (35.9)	0.013 **
	B	7 (14.9)	17 (43.6)	
	C	15 (31.9)	8 (20.5)	
Etiology	HBV	28 (59.6)	24 (61.5)	0.769 **
	HBV + HDV	3 (6.4)	4 (10.3)	
	Cryptogenic	7 (14.9)	4 (10.3)	
	HCV	6 (12.8)	6 (15.4)	
	Metabolic Disease	2 (4.3)	0 (0.0)	
	Ethanol	1 (2.1)	1 (2.6)	
LT Type	LDLT	47 (100)	37 (94.9)	0.203 ***
	DDLT	0 (0)	2 (5.1)	
Differentiation	Well	12 (25.5)	5 (12.8)	0.170 **
	Moderately	28 (59.6)	23 (59.0)	
	Poorly	7 (14.9)	11 (28.2)	
Vascular invasion	No	28 (59.6)	11 (28.2)	0.001 **
	Microvascular	19 (40.4)	21 (53.8)	
	Macrovascular	0 (0)	7 (18.0)	
Expanded Malatya Criteria	Within	47 (100)	14 (35.9)	<0.001 *
	Beyond	0 (0)	25 (64.1)	
PVTT	No	42 (89.4)	28 (71.8)	0.071 *
	Yes	5 (10.6)	11 (28.2)	
Outcomes	Alive	43 (91.5)	29 (74.4)	0.042 ***
	Exitus	4 (8.5)	10 (25.6)	
Recurrence	Absence	46 (97.9)	28 (71.8)	0.002 *
	Presence	1 (2.1)	11 (28.2)	
Percent of PCT stained tumoral area	No-staining	1 (2.13)	1 (2.6)	0.291 **
	<10%	4 (8.5)	5 (12.8)	
	10–25%	4 (8.5)	4 (10.3)	
	25–50%	4 (8.5)	9 (23.1)	
	>50%	34 (72.3)	20 (51.3)	
Intensity of PCT staining	No	1 (2.1)	2 (5.1)	0.579 **
	Weak	4 (8.5)	5 (12.8)	
	Moderate	9 (19.2)	10 (25.6)	
	Strong	33 (70.2)	22 (56.4)	

*: Chi-square test with Yates correction; **: Pearson chi-square; ***: Fisher’s exact chi-square test; HBV: Hepatitis B virus; HDV: Hepatitis D virus; HCV: Hepatitis C virus; LT: Liver transplantation; LDLT: Living donor liver transplantation; DDLT: Deceased donor liver transplantation; PVTT: Portal vein tumor thrombus.

**Table 5:** Analyses of quantitative variables in terms of the Expanded Malatya criteria.

Variables [Median (95%CI)]	Expanded Malatya Criteria		*p*-Value *
	Within	Beyond	
Age	58 (58–62)	56 (52–62)	0.977
BMI	27 (26–28)	26.6 (25–28)	0.655
MELD Score	12 (11–15)	11 (10–15)	0.333
Pretransplant last AFP level	11 (6–14)	45 (26–222)	<0.001
MTD	30 (30–50)	60 (40–80)	<0.001
Number of tumor nodules	1 (0–0)	4 (3–10)	<0.001
OS	1600 (1075–1824)	900 (545–1292)	0.006
DFS	1599 (1072–1822)	495 (376–1104)	0.001
Ki67 Staining (%)	5 (5–10)	10 (10–20)	0.010

* Mann–Whitney U-test; CI: Confidence Interval; BMI: Body mass index; MELD: Model For End-Stage Liver Disease; AFP: alpha fetoprotein; MTD: maximum dominant tumor diameter; OS: Overall survival; DFS: Disease-free survival.

**Table 6:** Analyses of qualitative variables in terms of the Expanded Malatya criteria.

Variables [n (%)]	Categories	Expanded Malatya Criteria		*p*-Value
		Within	Beyond	
Gender	Male	53 (86.9)	21 (84.0)	0.739 *
	Female	8 (13.1)	4 (16.0)	
Child Score	A	27 (44.3)	12 (48.0)	0.301 **
	B	15 (24.6)	9 (36.0)	
	C	19 (31.1)	4 (16.0)	
Etiology	HBV	34 (55.7)	18 (72.0)	0.658 **
	HBV + HDV	6 (9.8)	1 (4.0)	
	Cryptogenic	8 (13.1)	3 (12.0)	
	HCV	9 (14.75)	3 (12.0)	
	Metabolic disease	2 (3.3)	0 (0)	
	Ethanol	2 (3.3)	0 (0)	
LT Type	LDLT	61 (100)	23 (92.0)	0.082 *
	DDLT	0 (0)	2 (8.0)	
Differentiation	Well	13 (21.3)	4 (16.0)	0.021 **
	Moderately	40 (65.6)	11 (44.0)	
	Poorly	8 (13.1)	10 (40.0)	
Vascular invasion	No	35 (57.4)	4 (16.0)	<0.001 **
	Microvascular	26 (42.6)	14 (56.0)	
	Macrovascular	0 (0.0)	7 (28.0)	
Milan Criteria	Within	47 (77.1)	0 (0)	<0.001 ***
	Beyond	14 (22.9)	25 (100)	
PVTT	No	55 (90.2)	15 (60.0)	0.002 *
	Yes	6 (9.8)	10 (40.0)	
Outcomes	Alive	55 (90.2)	17 (68.0)	0.021 ***
	Exitus	6 (9.8)	8 (32.0)	
Recurrence	Absence	58 (95.1)	16 (64.0)	0.001 *
	Presence	3 (4.9)	9 (36.0)	
Percent of PCT stained tumoral area	No-staining	1 (1.6)	1 (4.0)	0.266 **
	<10%	7 (11.5)	2 (8.0)	
	10–25%	6 (9.8)	2 (8.0)	
	25–50%	6 (9.8)	7 (28.0)	
	>50%	41 (67.2)	13 (52.0)	
Intensity of PCT staining	No	1 (1.64)	2 (8.0)	0.367 **
	Weak	7 (11.5)	2 (8.0)	
	Moderate	12 (19.7)	7 (28.0)	
	Strong	41 (67.2)	14 (56.0)	

*: Fisher’s exact chi-square test; **: Pearson chi-square; ***: Chi-square test with Yates correction; HBV: Hepatitis B virus; HDV: Hepatitis D virus; HCV: Hepatitis C virus; LT: Liver transplantation; LDLT: Living donor liver transplantation; DDLT: Deceased donor liver transplantation; PVTT: Portal vein tumor thrombus.

**Table 7:** Analyses of quantitative variables in terms of outcomes.

Variables [Median (95%CI)]	Outcomes		*p*-Value *
	Alive	Dead	
Age	58 (58–62)	53 (48–63)	0.539
BMI	27 (25–28)	27 (25–28)	0.678
MELD Score	12 (11–15)	11 (9–12)	0.189
Pretransplant last AFP level	14 (9–24)	29 (13–195)	0.072
MTD (mm)	30 (30–40)	45 (30–80)	0.006
Number of tumor nodules	2 (2–3)	3 (1–11)	0.175
OS	1600 (1075–1807)	596 (215–900)	<0.001
DFS	1579 (1072–1786)	307 (201–523)	<0.001
Ki67 Staining (%)	5 (5–10)	15 (10–20)	0.003

* Mann–Whitney U-test; CI: Confidence Interval; BMI: Body mass index; MELD: Model For End-Stage Liver Disease; AFP: alpha fetoprotein; MTD: maximum dominant tumor diameter; OS: Overall survival; DFS: Disease free survival.

**Table 8:** Analyses of qualitative variables in terms of outcomes.

Variables [n (%)]	Categories	Outcomes		*p*-Value
		Alive	Dead	
Gender	Male	62 (86.1)	12 (85.7)	1.000 *
	Female	10 (13.9)	2 (14.3)	
Child Score	A	34 (47.2)	5 (35.7)	0.395 **
	B	18 (25.0)	6 (42.9)	
	C	20 (27.8)	3 (21.4)	
Etiology	HBV	44 (61.1)	8 (57.1)	0.446 **
	HBV + HDV	7 (9.7)	0 (0)	
	Cryptogenic	9 (12.5)	2 (14.3)	
	HCV	8 (11.1)	4 828.6)	
	Metabolic Disease	2 (2.8)	0 (0)	
	Ethanol	2 (2.8)	0 (0)	
LT Type	LDLT	71 (98.6)	13 (92.9)	0.301 *
	DDLT	1 (1.4)	1 (7.1)	
Differentiation	Well	15 (20.8)	2 (14.2)	0.088 **
	Moderately	45 (62.5)	6(42.9)	
	Poorly	12 (16.7)	6 (42.9)	
Vascular invasion	No	34 (47.2)	5 (35.7)	<0.001 **
	Microvascular	36 (50.0)	2 (28.6)	
	Macrovascular	2 (2.8)	5 (35.7)	
Milan Criteria	Within	43 (59.7)	4 (28.6)	0.042 ***
	Beyond	29 (40.3)	10 (71.4)	
Expanded Malatya Criteria	Within	55 (76.4)	6 (42.9)	0.027 ***
	Beyond	17 (23.6)	8 (57.1)	
PVTT	No	62 (86.1)	8 (57.1)	0.020 *
	Yes	10 (13.9)	6 (42.9)	
Recurrence	Absence	67 (93.1)	7 (50.0)	<0.001 *
	Presence	5 (6.9)	7 (50.0)	
Ki-67	≤5%	37 (51.4)	2 (14.3)	0.017 *
	>5%	35 (48.6)	12 (85.7)	
Percent of PCT stained tumoral area	No-staining	1 (1.4)	1 (7.1)	0.232 **
	<10%	9 (12.5)	0 (0)	
	10–25%	7 (9.7)	1 (7.1)	
	25–50%	9 (12.5)	4 (28.6)	
	>50%	46 (63.9)	8 (57.1)	
Intensity of PCT staining	No	2 (2.8)	1 (7.1)	0.740 **
	Weak	8 (11.1)	1 (7.1)	
	Moderate	15 (20.8)	4 (28.6)	
	Strong	47 (65.3)	8 (57.1)	

*: Fisher’s exact chi-square test; **: Pearson chi-square; ***: Chi-square test with Yates correction; HBV: Hepatitis B virus; HDV: Hepatitis D virus; HCV: Hepatitis C virus; LT: Liver transplantation; LDLT: Living donor liver transplantation; DDLT: Deceased donor liver transplantation; PVTT: Portal vein tumor thrombus.

## Data Availability

The datasets analyzed during the current study are available from the corresponding author on reasonable request.
